# MET Dependence Oversteps EGFR Dependence via Balancing Dimerization of the Receptor Tyrosine Kinases in Osimertinib‐Resistant *MET*‐Amplified, *EGFR*‐Mutated Non‐Small Cell Lung Cancer

**DOI:** 10.1111/1759-7714.70334

**Published:** 2026-06-30

**Authors:** Yuri Yagami, Takashi Sato, Hiroki Yamamoto, Hiromi Matsuo, Ryosuke Inoue, Ryouhei Tsutsumi, Mikiko Kakegawa, Yoshiro Nakahara, Jiichiro Sasaki, Katsuhiko Naoki

**Affiliations:** ^1^ Department of Respiratory Medicine Kitasato University School of Medicine Sagamihara Japan; ^2^ Department of Biochemistry Kitasato University School of Medicine Sagamihara Japan; ^3^ Research and Development Center for New Medical Frontiers Kitasato University School of Medicine Sagamihara Japan

**Keywords:** dimerization, drug resistance, *EGFR* mutation, lung cancer, *MET* amplification, osimertinib

## Abstract

**Background:**

Although epidermal growth factor receptor tyrosine kinase inhibitors (EGFR‐TKIs) have substantially improved the clinical outcomes of patients with *EGFR*‐mutated non‐small cell lung cancer (NSCLC), most patients ultimately develop acquired resistance. *MET* amplification is a common resistance mechanism to EGFR‐TKIs; however, its effects on receptor dimerization and the resulting dependence on EGFR or MET signaling have not been fully elucidated.

**Methods:**

We established a classical lung cancer cell line model with acquired resistance to osimertinib, a third‐generation EGFR‐TKI, through exposure to the drug (HCC827OR). Activation profiles of phospho‐receptor tyrosine kinases and genomic alterations were investigated, and drug sensitivity to osimertinib and the MET‐TKI savolitinib was evaluated. EGFR and MET signaling status and dimerization/interaction of EGFR, MET, and ERBB3 were analyzed. In addition, exogenous MET overexpression was introduced into parental HCC827 cells to assess its effects on drug sensitivity and receptor dimerization.

**Results:**

HCC827OR cells exhibited *MET* amplification and showed sensitivity to savolitinib monotherapy. ERBB3 phosphorylation correlated with the efficacy of the TKIs. Consistently, we found reduced interaction between EGFR and ERBB3 and increased interaction between MET and ERBB3 in HCC827OR cells. MET homodimers and MET‐EGFR interaction increased while EGFR homodimers decreased in those cells. Exogenous MET overexpression in the parental cells partially recapitulated the drug‐resistant phenotype, showing partial sensitivity to savolitinib, increased MET homodimers, and decreased EGFR homodimers.

**Conclusions:**

*MET* amplification alters the balance of EGFR/MET/ERBB3 dimerization, leading to a shift in signaling dependence from EGFR to MET. These findings provide insight into therapeutic strategies for *EGFR*‐mutated NSCLC.

## Graphical abstract

*MET* amplification in *EGFR*‐mutated NSCLC alters the balance of EGFR/MET/ERBB3 receptor dimerization, leading to a shift toward MET‐dominant signaling dependence during acquired resistance to osimertinib. Resistant cells exhibit decreased EGFR homodimers and EGFR‐ERBB3 interactions together with increased MET‐containing dimers, resulting in substantial growth suppression by MET inhibition. These findings provide insight into therapeutic strategies for *MET*‐amplified, *EGFR*‐mutated NSCLC.

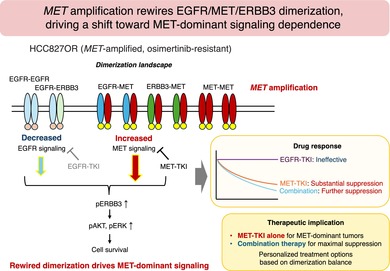

## 1 Introduction

Lung cancer is the most common cause of cancer‐related death worldwide [1, 2]. In non‐small cell lung cancer (NSCLC), which accounts for approximately 85% of lung cancer, molecular targeted therapeutics according to somatic gene alterations have been developed and have substantially improved the clinical outcomes. Among them, epidermal growth factor receptor (EGFR)‐tyrosine kinase inhibitors (TKIs) are effective against NSCLC with *EGFR*‐activating mutations, which are found in around half of lung adenocarcinoma [3]. However, acquired drug resistance eventually overcomes the efficacy of the drugs. Osimertinib, a third‐generation EGFR‐TKI effective against even NSCLC with secondary *EGFR* T790M resistance mutation, is not an exception.

Amplification of *MET*, the gene encoding the hepatocyte growth factor receptor, is a key EGFR‐independent resistance mechanism, found in 7%–15% of patients who develop clinical resistance to treatment with osimertinib [4]. Since EGFR and MET have downstream signaling pathways in common, such as the mitogen‐activated protein kinase (MAPK) pathway and the phospho‐inositide 3‐kinase (PI3K)‐AKT pathway, *MET* amplification allows MET to bypass EGFR signaling and mediate resistance to EGFR‐TKIs [5, 6]. It is speculated that *MET*‐amplified, *EGFR*‐mutated NSCLC is dependent on both EGFR and MET signaling, which can be treated with a combination of EGFR and MET inhibitors [5, 7]. In fact, clinical trials demonstrated that MET‐TKIs such as savolitinib, tepotinib, and capmatinib in combination with EGFR‐TKIs have antitumor activity in patients with *MET*‐amplified, *EGFR*‐mutated NSCLC [8, 9, 10]. Recently, amivantamab, a bi‐specific antibody that targets both EGFR and MET, in combination with chemotherapy, improved progression‐free survival compared with chemotherapy alone in *EGFR*‐mutated advanced NSCLC after disease progression on osimertinib [11]. Amivantamab plus lazertinib, another third‐generation EGFR‐TKI, also achieved longer progression‐free survival than osimertinib alone in even previously untreated *EGFR*‐mutated advanced NSCLC [12].

Resistance mechanisms to EGFR‐TKIs in *EGFR*‐mutated lung cancer have been explored by analyzing the models of drug‐resistant cell lines established through exposure to EGFR‐TKIs [5, 13, 14, 15]. One study reported *MET* amplification as a resistance mechanism to EGFR‐TKIs by investigating *EGFR*‐mutated NSCLC cell line HCC827 with acquired resistance to gefitinib, a first‐generation EGFR‐TKI, which was established through long‐term exposure to gefitinib with gradual dose escalation and was resistant to a single‐agent EGFR‐ or MET‐TKI but sensitive to the combination of both TKIs [5]. However, a recent study reported that a subset of *MET*‐amplified, *EGFR*‐mutated NSCLCs acquire dependence on MET alone and can be treated with a single‐agent MET‐TKI rather than the combination of EGFR and MET inhibitors [16]. While MET‐mediated activation of the kinase‐deficient HER family member ERBB3 led to resistance to the EGFR‐TKI in both studies [5, 16], the latter reported reduced *EGFR*:*MET* mRNA expression ratios in the MET‐dependent models [16]. Based on the previous findings, we hypothesized that the balance of EGFR/MET homodimerization and EGFR/MET/ERBB3 heterodimerization is associated with dependence on EGFR and/or MET in *MET*‐amplified, *EGFR*‐mutated NSCLC cells.

In this study, we made a classical model of the HCC827 cell line with acquired resistance to osimertinib (HCC827OR) through exposure to the drug, and the HCC827OR cells exhibited a shift toward MET dependence, showing partial sensitivity to the MET‐TKI savolitinib alone. We found that the balance of homo‐ and hetero‐dimerization of EGFR, MET, and ERBB3 was associated with MET‐dominant signaling dependence in the cancer cells. Our findings will provide additional evidence for the biology of *MET*‐amplified, *EGFR*‐mutated lung cancer.

## 2 Materials and Methods

### 2.1 Cell Lines and Reagents

HCC827 cells were from ATCC (CRL‐2868). Osimertinib‐resistant HCC827OR cells were established from HCC827 cells through exposure to osimertinib with stepwise escalation of drug concentration in our laboratory for 4 months. Both cell lines were maintained in RPMI‐1640 (Nacalai Tesque) supplemented with 10% FBS (Gibco) and 1% penicillin/streptomycin. Cells were regularly tested for mycoplasma using the mycoAlert Detection Kit (Lonza). Osimertinib and savolitinib were purchased from MedChemExpress. The cross‐linking reagent bis‐(sulfosuccinimidyl) suberate (BS3) was purchased from Thermo Fisher Scientific. Antibodies used in this study are listed in Table S1.

### 2.2 Cell Proliferation Assay

Cells were seeded onto 96‐well plates, and after an attachment period, cells were treated with the various concentrations of drugs for 72 h. Cell viability was measured using alamarBlue Cell Viability Reagent (Thermo Fisher Scientific) and fluorescence at 585 nm was measured on a Spectra Max3 220 plate reader (Molecular Devices) according to the manufacturer's protocol at excitation of 555 nm.

### 2.3 Phospho‐RTK Array Analysis

Tyrosine‐phosphorylated receptor tyrosine kinases (RTKs) were detected with the use of Proteome Profiler Array (R&D), which contains capture antibodies to 49 RTKs in duplicate wells. Cell lysates were incubated overnight at 4°C with the array in the provided buffer. Target RTKs were captured by the respective capture antibodies, and tyrosine‐phosphorylated RTKs were subsequently detected with horseradish peroxidase‐conjugated antibodies to phosphotyrosine.

### 2.4 Western Blotting and Coimmunoprecipitation

To determine protein expression, cells were lysed in lysis buffer (40 mM Tris‐HCl pH 8.0, 150 mM NaCl, 2 mM EDTA, 1% IGEPAL CA‐630, 0.5% Na deoxycholate) with Protease/Phosphatase inhibitor cocktail (R&D). After centrifugation to remove insoluble debris, cell lysates were separated by electrophoresis on an SDS–polyacrylamide gel electrophoresis gel (ATTO) and transferred to polyvinylidene difluoride membranes (Merck), which were blocked for 1 h with TBS + 0.1% Tween 20 with 5% milk (w/v) or 5% BSA. The membranes were immunoblotted with use of primary antibodies overnight at 4°C. The proteins were visualized using Fusion Fx (Vilber).

For coimmunoprecipitation, cells were lysed in lysis buffer (25 mM Tris‐HCl pH 7.4, 150 mM NaCl, 1 mM EDTA, 5% glycerol, 1.0% IGEPAL CA‐630, 0.5% Na‐deoxycholate) with Protease inhibitor cocktail. The lysates were incubated overnight at 4°C with anti‐EGFR, anti‐ERBB3, or an irrelevant IgG, and subsequently for an hour with Dynabeads (Thermo Fisher Scientific). After washing with PBS + 0.05% Tween 20, the beads were boiled for 10 min in 1× SDS sample buffer. The immunoprecipitates were then processed for immunoblotting.

Band intensities were quantified using ImageJ software Ver. 1.54 g and normalized to parental controls. Statistical significance was assessed using a one‐sample ratio *t*‐test against a hypothetical value of 1.

### 2.5 Cross‐Linking of EGFR and MET

Prior to cross‐linking, the cells were subjected to 2 mM of the cross‐linking reagent BS3 for 1 h at 4°C. The reaction was quenched using 10 mM Tris for 15 min at room temperature. Subsequently, cells were concentrated for 4 min and solubilized in lysis buffer containing 40 mmol/L Tris‐HCl pH 8.0, 150 mmol/L NaCl, 2 mmol/L EDTA, 1% IGEPAL CA‐630, 0.5% Na deoxycholate, and protease inhibitors.

### 2.6 Whole Exome Sequencing Analysis

Genomic DNA was extracted using the DNeasy Blood & Tissue Kit (Qiagen). Whole‐exome sequencing (WES) was performed by Macrogen Inc. Exome capture was conducted using the SureSelect^XT^ Human All Exon V6 Library kit (Agilent). Sequencing was performed on NovaSeq6000 (Illumina) for 150 nucleotides each from paired end. Cleaned fastq files were mapped onto the human reference genome version GRCh38 (hg38) using BWA‐MEM version 0.7.17. Single nucleotide variations, insertions and deletions were detected using Genome Analysis Toolkit version 4.0.5.1. Filtered variants are annotated with SnpEff 4.3t (build 2017‐11‐24) and filtered with dbSNP and SNPs from the 1000 genome project. Copy number variations were inferred from the sequencing data using CNVkit and ExomeCNV.

### 2.7 Overexpression of MET

To generate MET‐overexpressing cell lines, HCC827 or GFP open reading frame was cloned into pLEX_306 (a gift from Dr. David Root, Addgene #41392) using Gateway cloning methods according to the manufacturer's recommendations. HEK293T cells were seeded in a 10‐cm tissue culture dish and incubated at 37°C and 5% CO_2_ in order to produce lentiviral vectors. To construct the vector, the MET sequence (NM_000245) was used. Cells were transduced by lentiviral infection and selected with puromycin for 5 days.

### 2.8 Proximity Ligation Assay (PLA)

Cells (3.0 × 10^4^) were seeded on poly‐L‐lysine‐coated 12‐mm circular glass coverslips for 24 h, fixed in 4% paraformaldehyde/PBS for 10 min at RT, permeabilized with 0.1% Triton X‐100, 0.2% BSA/PBS for 10 min, and blocked with 1% BSA/PBS for 30 min. Fixed cells were incubated with primary antibodies as indicated, and then treated with donkey anti‐mouse (Duolink In Situ PLA Probe Anti‐Mouse MINUS, Sigma) and donkey anti‐rabbit (Duolink In Situ PLA Probe Anti‐Rabbit PLUS, SIGMA) secondary antibodies, followed by PLA procedure according to the manufacturer's protocol. Coverslips were then mounted on glass slides using Prolong Diamond antifade mountant containing DAPI (Thermo Fisher). Images were obtained using an LSM980 confocal microscopy system with Zen 3.9 as unsaturated 16‐bit TIFF images. The confocal microscopes were set to obtain approximately 2 Airy Unit (AU), and single‐plane images are presented in figures. The numbers of punctate signals generated by PLA and the numbers of nuclei in images were counted using the “Find Maxima” function and the “Analyze Particles” function of FIJI/ImageJ software Ver. 1.54p, respectively, from 5 images per condition. Statistical significance was assessed using two‐tailed Welch's *t*‐test.

## 3 Results

### 3.1 *MET* Is Amplified in HCC827OR Cells

**FIGURE 1:**
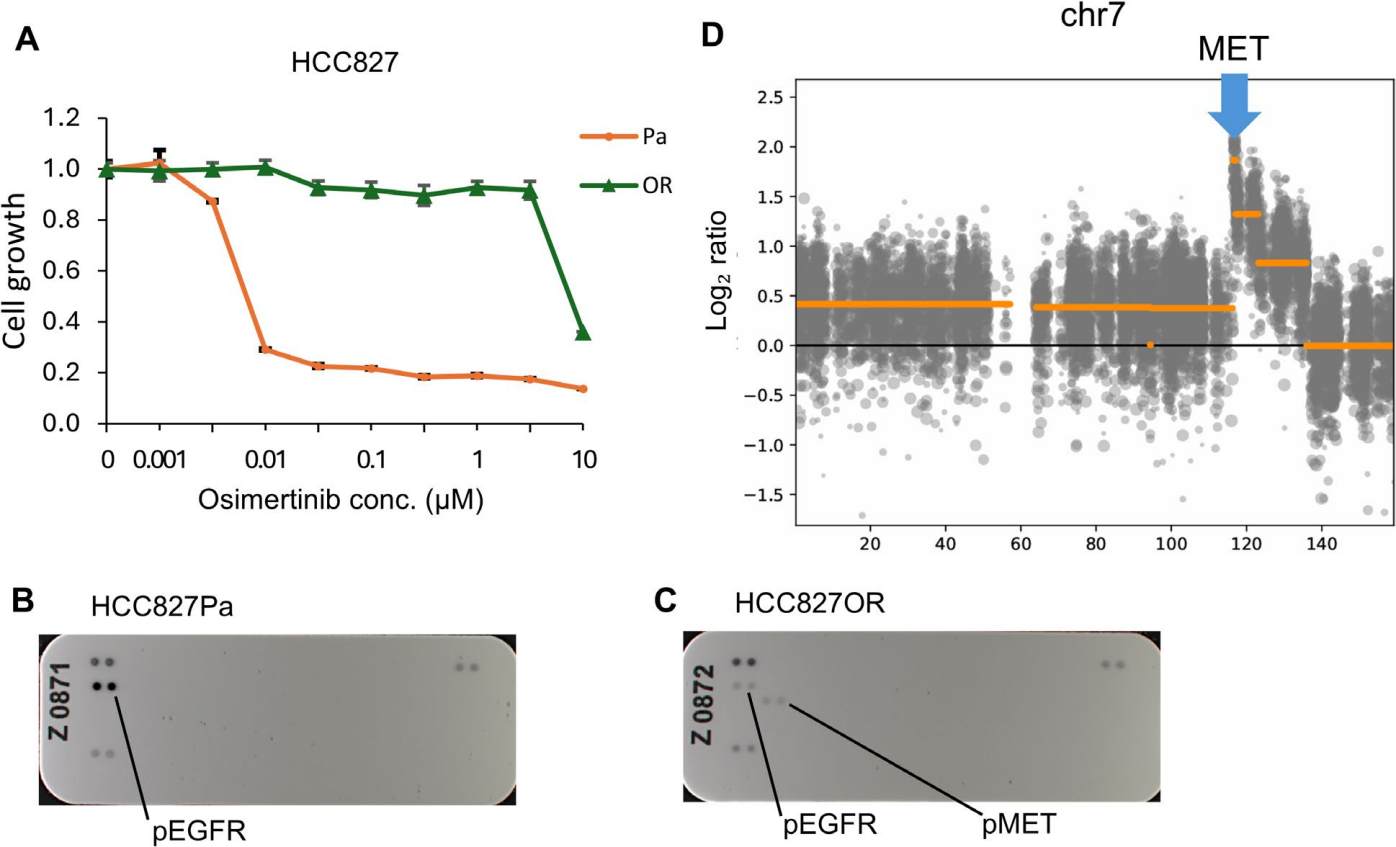
Characteristics of HCC827 osimertinib resistant (OR) cells. (A) Cell growth of HCC827 parental cells (Pa) and HCC827OR cells was assessed after addition of osimertinib for 72 h. (B, C) Human phospho‐receptor tyrosine kinase array was performed for HCC827Pa (B) and HCC827OR (C) cells. (D) Log ratio of copy number variations at chromosome 7 in HCC827OR cells compared with HCC827Pa cells.

We first confirmed that HCC827OR cells were not responsive to osimertinib even at the concentration of 1 μM (Figure 1A). Since activation of other receptor tyrosine kinases such as the ERBB family, MET, and FGFR has been found as the mechanism of EGFR‐TKI resistance in EGFR‐mutated lung cancer [4, 5, 14, 15], we investigated phospho‐RTK activation profiles of HCC827Pa and HCC827OR cells. In HCC827Pa cells, high signals of phosphorylated EGFR were detected as expected (Figure 1B). In contrast, in HCC827OR cells, we found phosphorylation of MET in addition to phosphorylation of EGFR (Figure 1C).

To investigate whether additional genomic alterations caused the resistance to osimertinib, we next performed whole exome sequencing on HCC827Pa and HCC827OR cells (Figure 1D). While HCC827OR cells maintained the *EGFR* del E756_A750 mutation, we did not find other mutations in HCC827OR cells that could lead to the resistance to osimertinib (Table S2). When we investigated copy number variation on these cells, copy number gain at the *MET* locus in HCC827OR cells was detected (Figure 1D, Table S3A,B). Since *MET* amplification is a known mechanism of resistance to EGFR‐TKI in *EGFR*‐mutant lung cancer cells [5], we suspected that *MET* amplification caused resistance to osimertinib in our model.

### 3.2 Inhibition of MET Alone Suppresses the Growth of HCC827OR Cells

**FIGURE 2:**
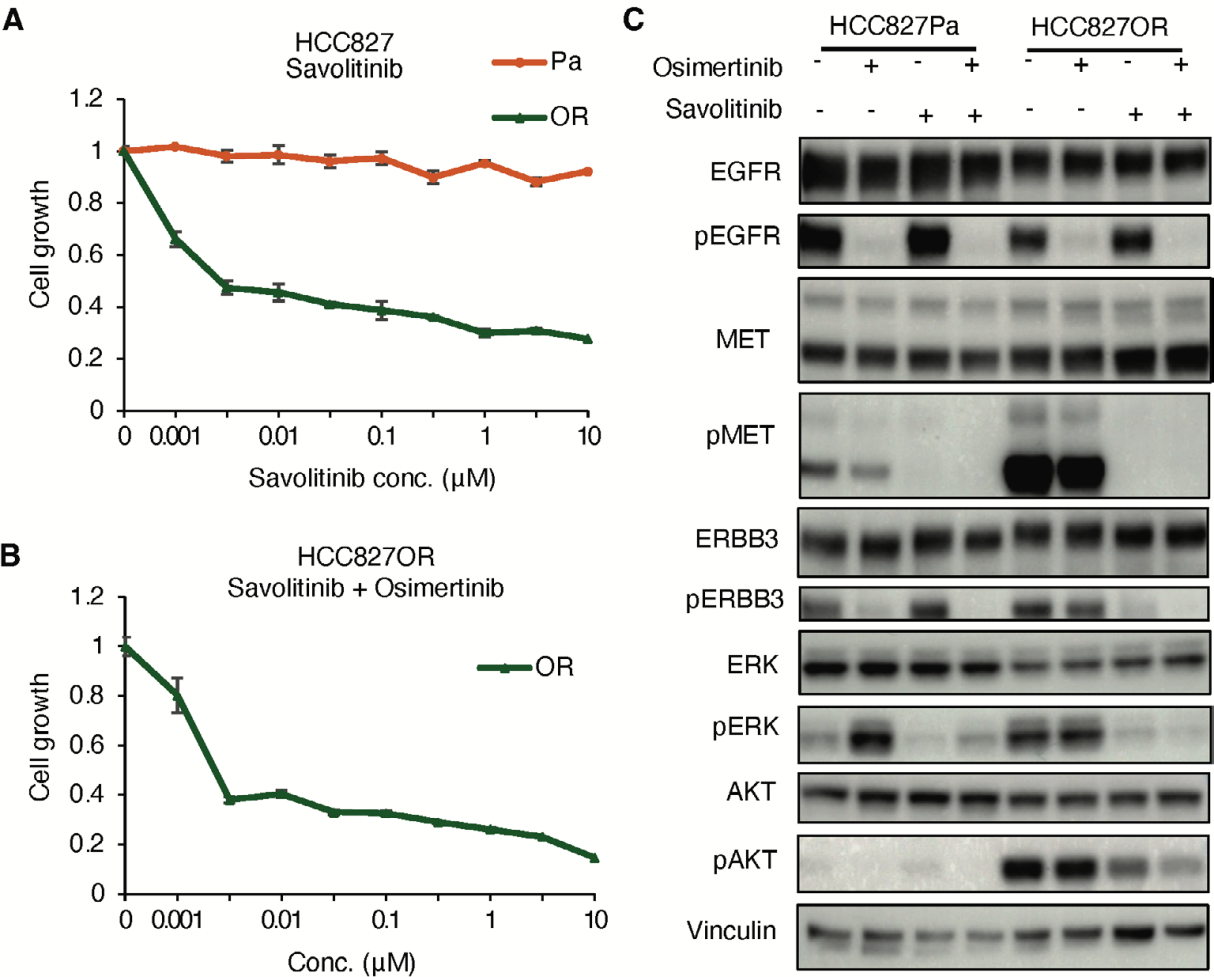
Inhibition of MET alone suppresses the growth of HCC827OR cells and the downstream signaling. (A) Cell growth of HCC827Pa and HCC827OR cells was assessed after addition of savolitinib for 72 h. (B) Cell growth of HCC827OR cells was assessed after addition of the combination of savolitinib and osimertinib for 72 h. (C) Effects of osimertinib and savolitinib on signals downstream of EGFR/MET/ERBB3 in HCC827Pa and HCC827OR cells were assessed by immunoblotting. Vinculin was used as a loading control. Cells were treated with 1 μM osimertinib alone, 1 μM savolitinib alone, or both drugs in combination for 4 h.

In order to investigate whether increased MET signaling is responsible for the acquired resistance to osimertinib, we treated HCC827OR cells with savolitinib, a MET‐TKI, alone or in combination with osimertinib (Figure 2A,B, Figure S1). It is noteworthy that savolitinib alone suppressed cell growth in HCC827OR cells without osimertinib (Figure 2A) although osimertinib additionally suppressed cell growth of HCC827OR cells under exposure to savolitinib (Figure 2B, Figure S1). This suggests that *MET* amplification not only led to resistance to osimertinib but also weakened dependence on EGFR signaling in HCC827OR cells while these cells retain *EGFR* mutation (Table S2). By western blotting (Figure 2C), both total MET and phospho‐MET protein levels were higher in HCC827OR cells compared with those in HCC827Pa cells. Second, phosphorylation of MET, ERBB3, ERK, and AKT was reduced by savolitinib alone in HCC827OR cells while EGFR phosphorylation was sustained by savolitinib alone. By the combination of savolitinib and osimertinib, phosphorylation of EGFR, MET, ERBB3, ERK, and AKT was entirely suppressed in HCC827OR cells. To investigate whether the other RTKs were activated via exposure to savolitinib, we examined phospho‐RTK activation profiles of HCC827Pa and HCC827OR cells that were exposed to savolitinib (Figure S2) and detected phosphorylated ERBB3 in addition to phosphorylated EGFR in HCC827Pa cells and weak signals of phosphorylated EGFR in HCC827OR cells. These results suggest that the resistance to osimertinib and acquired dependence on MET in HCC827OR cells are caused by increased MET signaling and interactions among EGFR, MET, and ERBB3 proteins.

### 3.3 EGFR Homodimers and Heterodimers With ERBB3 Decrease While MET Homodimers and Heterodimers With EGFR and ERBB3 Increase in HCC827OR Cells

**FIGURE 3:**
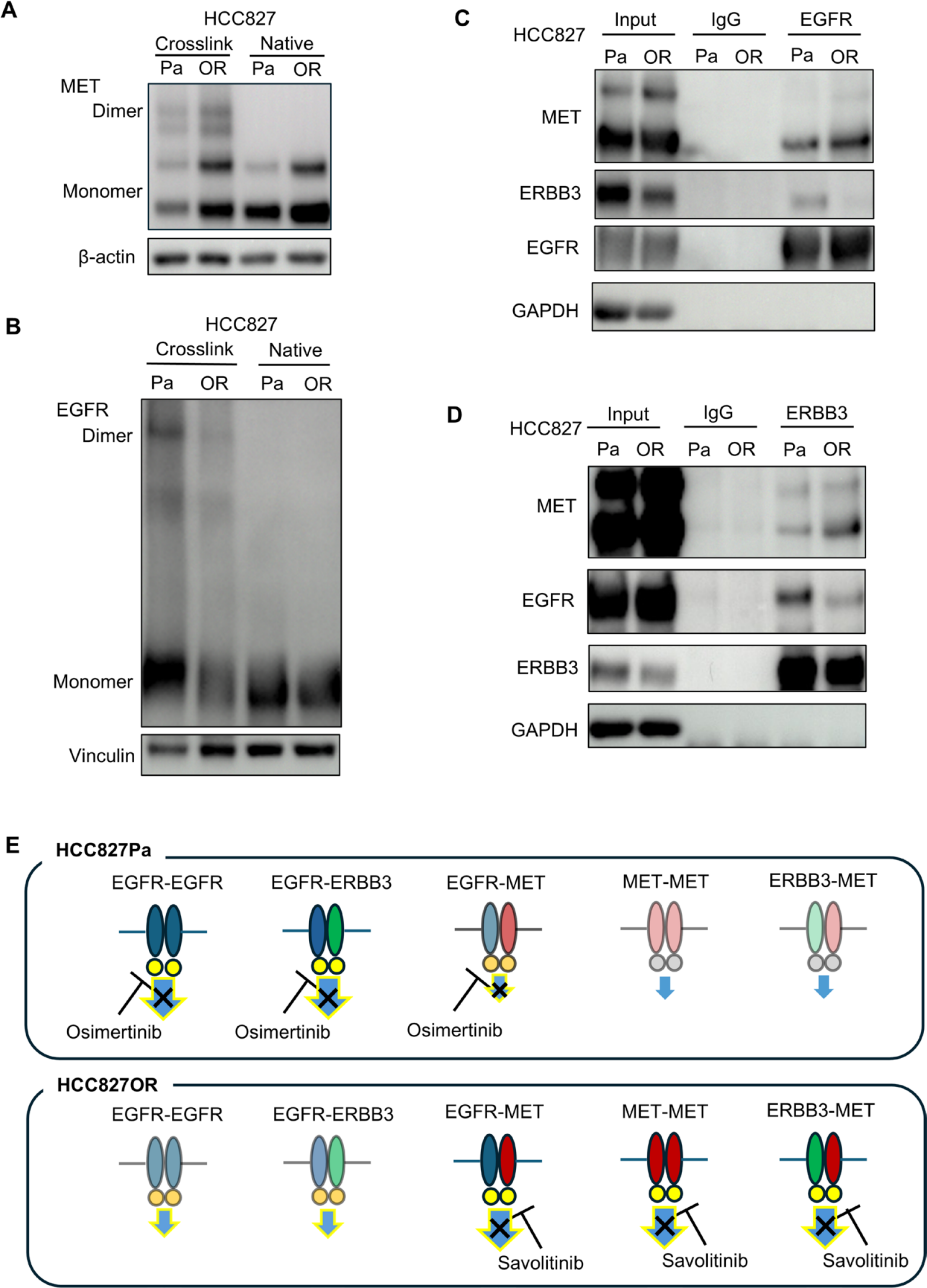
EGFR homodimers and heterodimers with ERBB3 decrease while MET homodimers and heterodimers with EGFR and ERBB3 increase in HCC827OR cells. (A) Detection of MET by immunoblotting in HCC827Pa and HCC827OR cells with or without chemical crosslinking. β‐Actin was used as loading control. (B) Detection of EGFR by immunoblotting in HCC827Pa and HCC827OR cells with or without chemical crosslinking. Vinculin was used as loading control. (C) EGFR‐MET and EGFR‐ERBB3 interaction shown by coimmunoprecipitation of MET and ERBB3 using an antibody against endogenous EGFR in HCC827Pa and HCC827OR cells. GAPDH was used as loading control. (D) ERBB3‐EGFR and ERBB3‐MET interaction shown by coimmunoprecipitation of EGFR and MET using an antibody against endogenous ERBB3 in HCC827Pa and HCC827OR cells. GAPDH was used as loading control. (E) Schematic images of receptor dimerization in HCC827Pa and HCC827OR cells.

Next, we investigated the interaction among EGFR, MET and ERBB3 proteins by assessing their dimerization status in HCC827Pa and HCC827OR cells. By chemical crosslinking of EGFR and MET, we found that the amount of MET homodimers in HCC827OR cells tended to be larger than that in HCC827Pa while EGFR homodimers decreased in HCC827OR cells (Figure 3A,B, Figure S3A,B). Additionally, by endogenous coimmunoprecipitation, we found that the amount of MET interacting with EGFR and ERBB3 in HCC827OR cells was larger than that in HCC827Pa cells (Figure 3C,D, Figure S3C,D) while the amount of ERBB3 interacting with EGFR in HCC827OR cells was smaller than that in HCC827Pa cells (Figure 3C, Figure S3E), indicating that MET heterodimers with EGFR and ERBB3 increased and EGFR‐ERBB3 heterodimers decreased in HCC827OR cells. PLA experiments further supported increased MET‐ERBB3 heterodimers and decreased EGFR‐ERBB3 heterodimers in HCC827OR cells (Figure S4). These findings suggest that HCC827OR cells acquire dependence on MET signaling and, by contrast, diminish dependence on EGFR signaling through increase of MET homodimers and heterodimers with EGFR and ERBB3 and decrease of EGFR homodimers and EGFR‐ERBB3 heterodimers (Figure 3E).

### 3.4 Overexpression of MET in HCC827Pa Partially Mimics the HCC827OR Model

**FIGURE 4:**
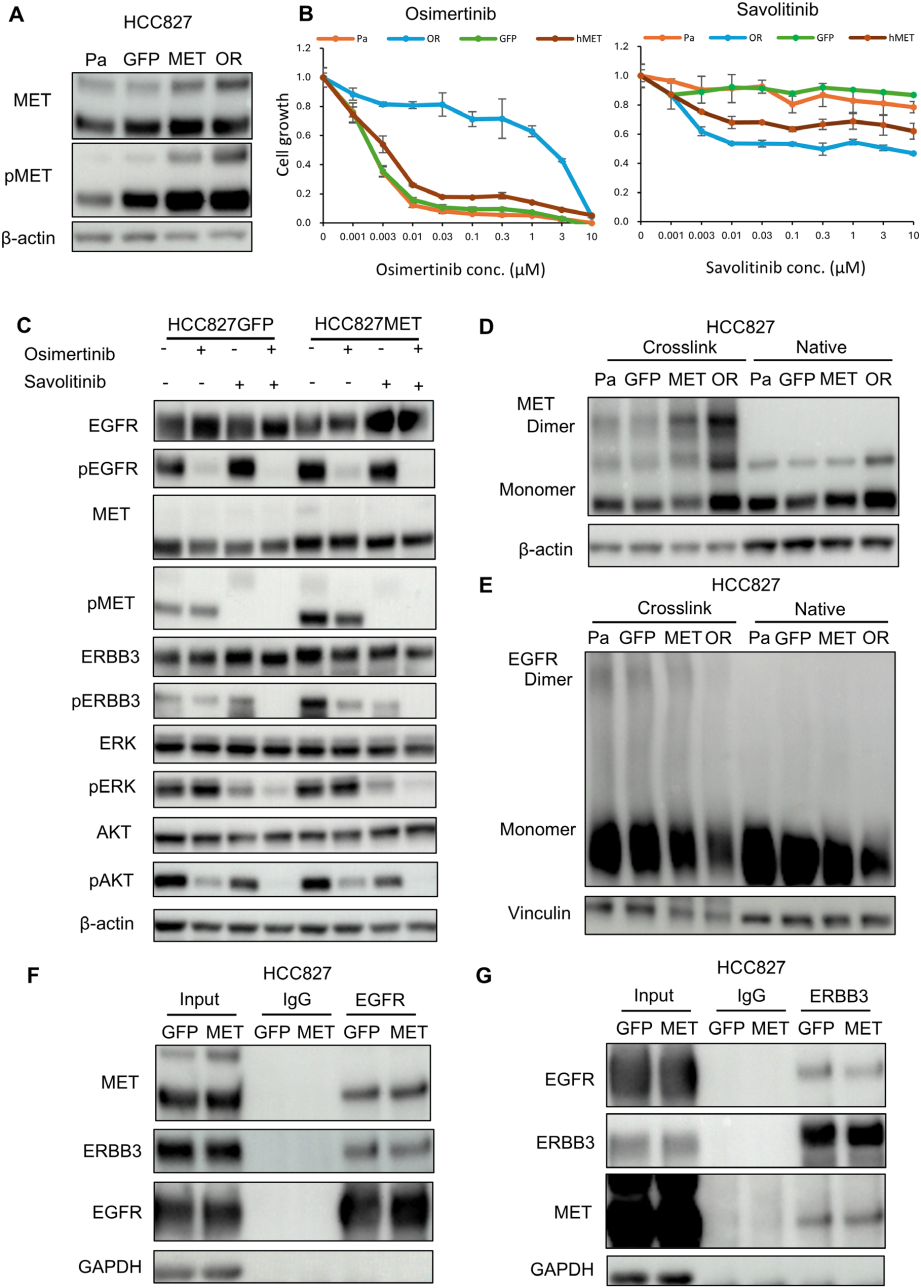
Met overexpression in HCC827Pa cells partially mimics the HCC827OR model with increased MET‐MET/MET‐EGFR dimers and decreased EGFR‐EGFR dimers. (A) Protein expression of MET, phospho‐MET, and β‐Actin as a loading control in parental (Pa), GFP‐overexpressed (GFP), MET‐overexpressed (MET) HCC827 cells and HCC827OR cells. (B) Cell growth of HCC827Pa, GFP, MET, and OR cells was assessed after addition of osimertinib (*left*) or savolitinib (*right*) for 72 h. (C) Effects of osimertinib and savolitinib on signals downstream of EGFR/MET/ERBB3 in HCC827GFP and HCC827MET cells were assessed by immunoblotting. β‐Actin was used as a loading control. Cells were treated with 1 μM osimertinib alone, 1 μM savolitinib alone, or both drugs in combination for 4 h. (D) Detection of MET by immunoblotting in HCC827Pa, GFP, MET, and OR cells with or without chemical crosslinking. β‐Actin was used as a loading control. (E) Detection of EGFR by immunoblotting in HCC827Pa, GFP, MET, and OR cells with or without chemical crosslinking. Vinculin was used as a loading control. (F) EGFR‐MET and EGFR‐ERBB3 interaction shown by coimmunoprecipitation of MET and ERBB3 using an antibody against endogenous EGFR in HCC827GFP and HCC827MET cells. GAPDH was used as a loading control. (G) ERBB3‐EGFR and ERBB3‐MET interaction shown by coimmunoprecipitation of EGFR and MET using an antibody against endogenous ERBB3 in HCC827Pa and HCC827OR cells. GAPDH was used as a loading control.

To investigate whether increased amount of MET protein leads to acquired resistance to EGFR‐TKI and the relative MET dependence on MET in *EGFR*‐mutated lung cancer cells, we exogenously overexpressed MET in HCC827Pa cells (Figure 4A). We found that MET‐overexpressed HCC827 cells (HCC827MET) showed mild resistance to osimertinib and weak sensitivity to savolitinib (Figure 4B). When we examined total‐ and phospho‐protein levels of the RTK signaling in HCC827MET cells (Figure 4C), firstly, total MET and phospho‐MET protein levels were higher in HCC827MET cells compared with those in control HCC827 cells. Second, savolitinib alone substantially reduced phosphorylation of MET and further reduced phosphorylation of ERBB3, ERK and AKT partially in HCC827MET cells. Osimertinib alone also reduced phosphorylation of ERBB3 and AKT, compatible with their remaining sensitivity to osimertinib alone. Combination of osimertinib and savolitinib further reduced phosphorylation of EGFR, MET, ERBB3, ERK and AKT. While HCC827MET cells exhibited larger amount of MET homodimers and smaller amount of EGFR homodimers compared with control HCC827 cells, we did not find significant increase of interaction of MET with EGFR nor ERBB3 nor decrease of interaction between EGFR and ERBB3 in HCC827MET cells (Figure 4D–G). These results suggest that MET overexpression partially mimics our HCC827OR model through the increase of MET‐MET and EGFR‐MET dimers.

## 4 Discussion

In this study, we demonstrated that *MET* amplification in *EGFR*‐mutated NSCLC cells with acquired resistance to an EGFR‐TKI alters the balance of receptor dimerization, characterized by increased MET‐containing dimers and decreased EGFR homodimers and heterodimers with ERBB3. This reorganization leads to a shift toward MET‐dominant signaling dependence rather than sole dependence. Notably, in contrast to the classical bypass model [5], in which MET inhibition alone has minimal effect, our model shows substantial growth suppression with MET‐TKI monotherapy, supporting a shift in signaling dependency rather than a purely bypass‐driven mechanism. Our findings provide insight into the understanding of *MET*‐amplified, *EGFR*‐mutated NSCLC and how to make therapeutic strategies against *EGFR*‐mutated NSCLC considering *MET* amplification as a resistance mechanism to EGFR‐TKIs.

*MET* amplification is a common resistance mechanism to EGFR‐TKIs in *EGFR*‐mutated NSCLC, which was first described in a drug‐resistant HCC827 cell line model using gefitinib, a first‐generation EGFR‐TKI [5]. In this model, *MET*‐amplified, *EGFR*‐mutated cells were co‐dependent on EGFR and MET and were resistant to either EGFR‐ or MET‐TKI alone but sensitive to the combination of both TKIs. *MET* amplification led to activation of ERBB3, causing the resistance to gefitinib in those cells. In this study, although we developed osimertinib‐resistant cell line from the same parental lung cancer cell line HCC827, our model showed growth suppression with the MET‐TKI alone. Not only phosphorylation of MET but also phosphorylation of ERBB3 and the downstream AKT and ERK were inhibited by a MET‐TKI alone. In the recent PDX models of *MET*‐amplified, *EGFR*‐mutated NSCLC showing sole MET dependence, *EGFR*:*MET* transcript ratios were reported to be lower compared with the previous model showing co‐dependence on EGFR and MET [16]. In *MET*‐amplified lung cancer cell lines without *EGFR* mutation, EGFR and ERBB3 form heterodimers with MET, which were disassociated by a MET‐TKI, and RNA interference‐mediated depletion of EGFR or ERBB3 inhibited cell proliferation less effectively compared with MET depletion [17]. Based on these pieces of knowledge, we focused on EGFR/MET homo‐ and EGFR/MET/ERBB3 heterodimerization in this study and found that our HCC827OR cells harbored more MET homodimers and heterodimers with ERBB3 and EGFR and less EGFR homodimers and heterodimers with MET and ERBB3 compared with HCC827Pa cells, suggesting that the balance of those dimers contributes to the dependence on EGFR and/or MET in *MET*‐amplified, *EGFR*‐mutated cells. Particularly, interaction of ERBB3 with EGFR or MET reflected the efficacy of MET‐TKI in those cells, suggesting that ERBB3 heterodimers preferentially contribute to cell survival and sensitivities to the TKIs compared to EGFR and MET homodimers. This finding is supported by a previous report demonstrating that EGFR‐ERBB3 heterodimers are more potent at transducing growth and survival signals than EGFR homodimers [18].

We further overexpressed MET in HCC827Pa cells to see whether increased MET expression led to osimertinib resistance and MET dependence similarly as our *MET*‐amplified HCC827OR cells. As a result, HCC827MET cells conferred both sensitivity to savolitinib and resistance to osimertinib partially. The MET‐TKI alone suppressed not only phosphorylation of MET but also phosphorylation of the downstream ERK, AKT, and ERBB3 to some extent in the HCC827MET cells, which was consistent with the degree of their dependence on EGFR and MET. A potential explanation why HCC827MET cells remain sensitive to osimertinib is that the amount of ERBB3 heterodimers with MET did not increase in these cells unlike in HCC827OR cells. These results reflected that cell proliferation of HCC827MET was suppressed partially under exposure to MET‐TKI.

Our findings will provide implications for the therapeutic strategies against *EGFR*‐mutated NSCLC. *MET*‐amplified, *EGFR*‐mutated tumors with predominant MET dependence could be treated with a MET‐TKI alone while those with co‐dependence on EGFR and MET need inhibition of both EGFR and MET. Some of the therapeutic strategies against *EGFR*‐mutated NSCLCs in recent clinical trials have relied on concurrent inhibition of EGFR and MET [11, 12, 19], however, those tumors may not always need concurrent inhibition of the RTKs considering the toxicity of both EGFR and MET inhibitors and possibly the introduction of further drug resistance to both inhibitors. There's still room for optimization of the therapeutics, which warrants further investigations [20]. Further validation using clinical specimens will be important to determine whether dimerization patterns can stratify *MET*‐amplified, *EGFR*‐mutated tumors according to their signaling dependence and therapeutic vulnerability.

Taken together, we demonstrated that our *MET*‐amplified, *EGFR*‐mutated NSCLC cells resistant to osimertinib exhibited a shift toward MET‐dominant dependence, remaining treatable with a MET‐TKI alone. The dependence was associated with the balance of homo‐ and heterodimerization of EGFR, MET, and ERBB3. Optimized therapeutic strategies in consideration of dependence in each tumor may be warranted.

## Author Contributions

**Takashi Sato:** conceptualization, methodology, funding acquisition, project administration, supervision, writing – original draft, writing – review and editing, formal analysis. **Hiroki Yamamoto:** investigation, writing – review and editing. **Yuri Yagami:** conceptualization, formal analysis, investigation, visualization, writing – original draft, project administration. **Mikiko Kakegawa:** conceptualization, investigation, writing – review and editing. **Ryouhei Tsutsumi:** methodology, investigation, writing – review and editing, formal analysis. **Ryosuke Inoue:** investigation, writing – review and editing. **Yoshiro Nakahara:** conceptualization, writing – review and editing. **Katsuhiko Naoki:** conceptualization, funding acquisition, supervision, writing – review and editing. **Jiichiro Sasaki:** conceptualization, methodology, writing – review and editing. **Hiromi Matsuo:** formal analysis, investigation, writing – review and editing, visualization.

## Funding

This work was supported by Japan Society for the Promotion of Science, 23K07609, 22K08288; Naito Foundation.

## Conflicts of Interest

The authors declare no conflicts of interest.

## Supporting information


**Figure S1:** Cell growth of HCC827Pa and HCC827OR cells was assessed after addition of osimertinib with 1 μM savolitinib for 72 h.

**Figure S2:** Human phospho‐receptor tyrosine kinase array was performed for HCC827Pa and HCC827OR cells after treatment with savolitinib for 4 h.

**Figure S3:** Relative amounts of MET dimers (A) and EGFR dimers (B) in HCC827OR cells compared with HCC827Pa cells were quantified from immunoblots obtained with or without chemical crosslinking (*n* = 3, respectively). Relative EGFR‐MET (C), ERBB3‐MET (D), and ERBB3‐EGFR (E) interactions in HCC827OR cells compared with HCC827Pa cells were quantified from coimmunoprecipitation immunoblots (*n* = 3, respectively).

**Figure S4:** Cells were subjected to PLA (left) with indicated pairs of antibodies (gray). Nuclei were stained with DAPI (blue). Representative images from one of 3 independent replicates are shown. Scale bar: 50 μm. Average numbers of punctate PLA signals per cell were counted from 5 images, and plotted in the graphs (right).

**Table S1:** Details of the antibodies used in this study.

**Table S2:** Single nucleotide variations, insertions and deletions detected in HCC827Pa and HCC827OR cells.

**Table S3:** (A) Copy number variations in HCC827Pa and HCC827OR cells. (B) Copy number comparison between HCC827Pa and HCC827OR cells. copy. number: CNV call. 1 = deletion, 2 = normal, 3 and more = amplification.

## Data Availability

Data will be available upon reasonable request to the corresponding author.
