# Supplementary figures and images for: MET Dependence Oversteps EGFR Dependence via Balancing Dimerization of the Receptor Tyrosine Kinases in Osimertinib‐Resistant MET‐Amplified, EGFR‐Mutated Non‐Small Cell Lung Cancer
Source: Thorac Cancer. 2026 Jun 30;17(13):e70334. doi: 10.1111/1759-7714.70334 (PMC13319413; doi:10.1111/1759-7714.70334)

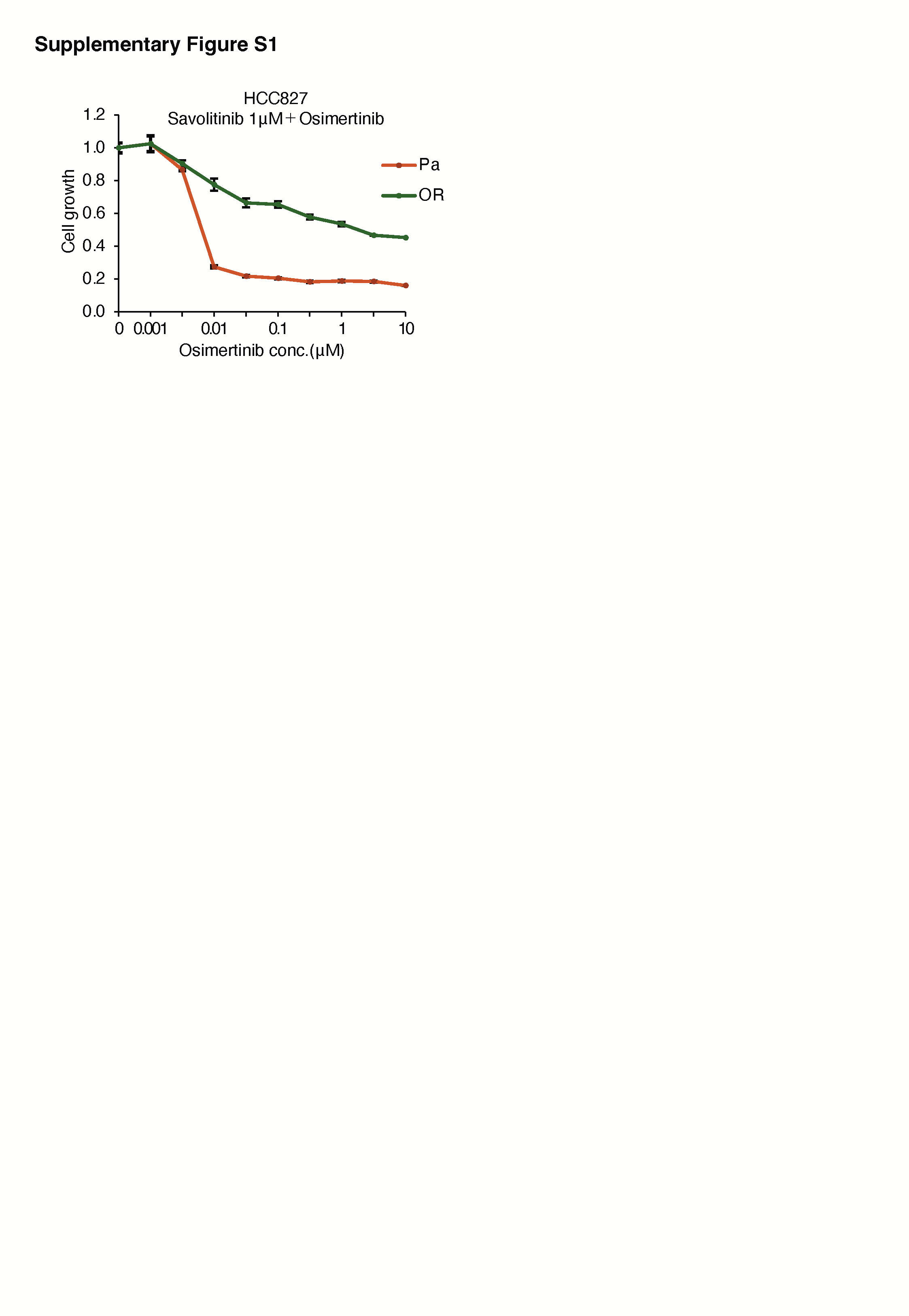

Supplement: Supplementary file 1 — Figure S1: Cell growth of HCC827Pa and HCC827OR cells was assessed after addition of osimertinib with 1 μM savolitinib for 72 h. [file TCA-17-e70334-s003.tiff]

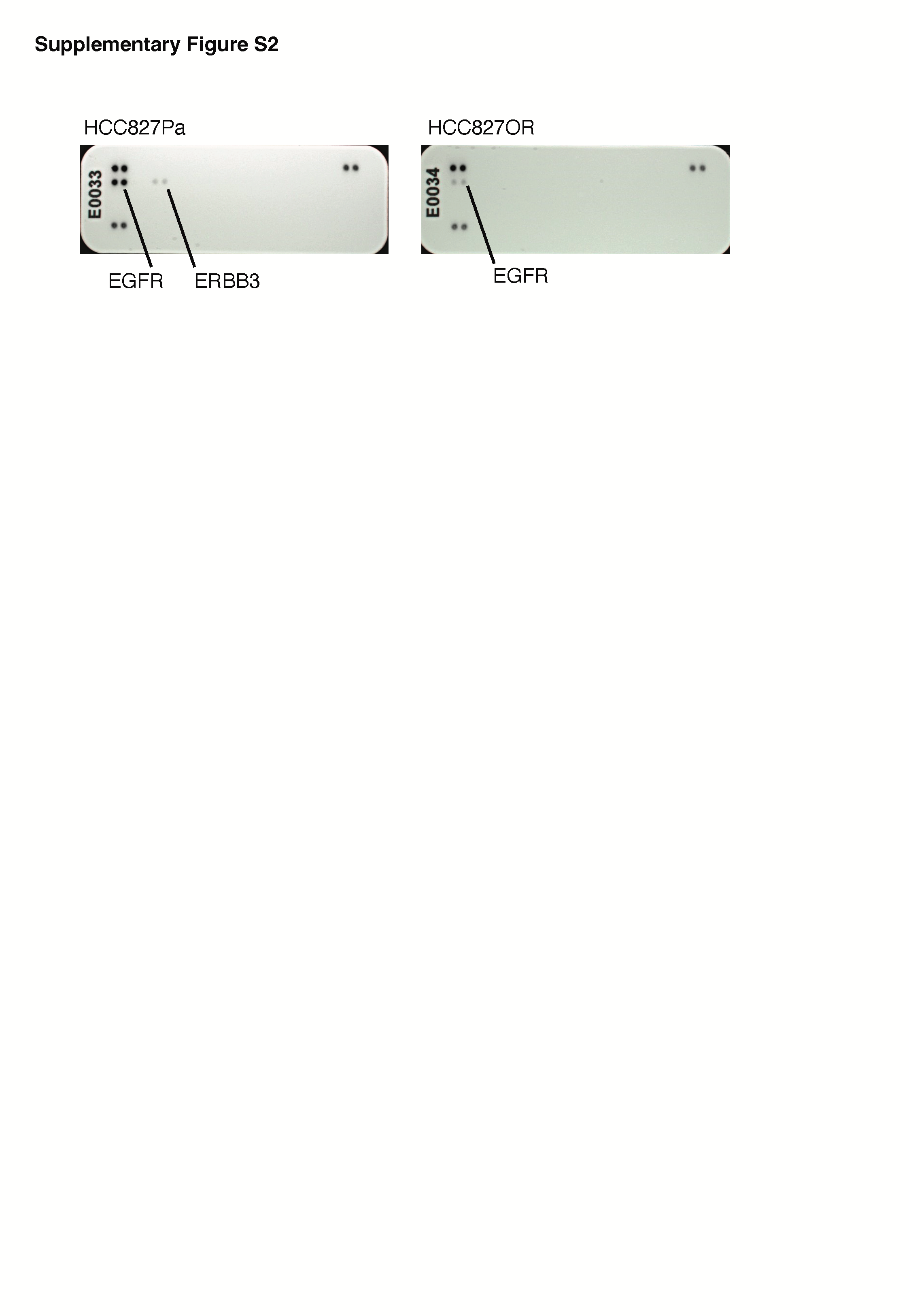

Supplement: Supplementary file 2 — Figure S2: Human phospho‐receptor tyrosine kinase array was performed for HCC827Pa and HCC827OR cells after treatment with savolitinib for 4 h. [file TCA-17-e70334-s006.tiff]

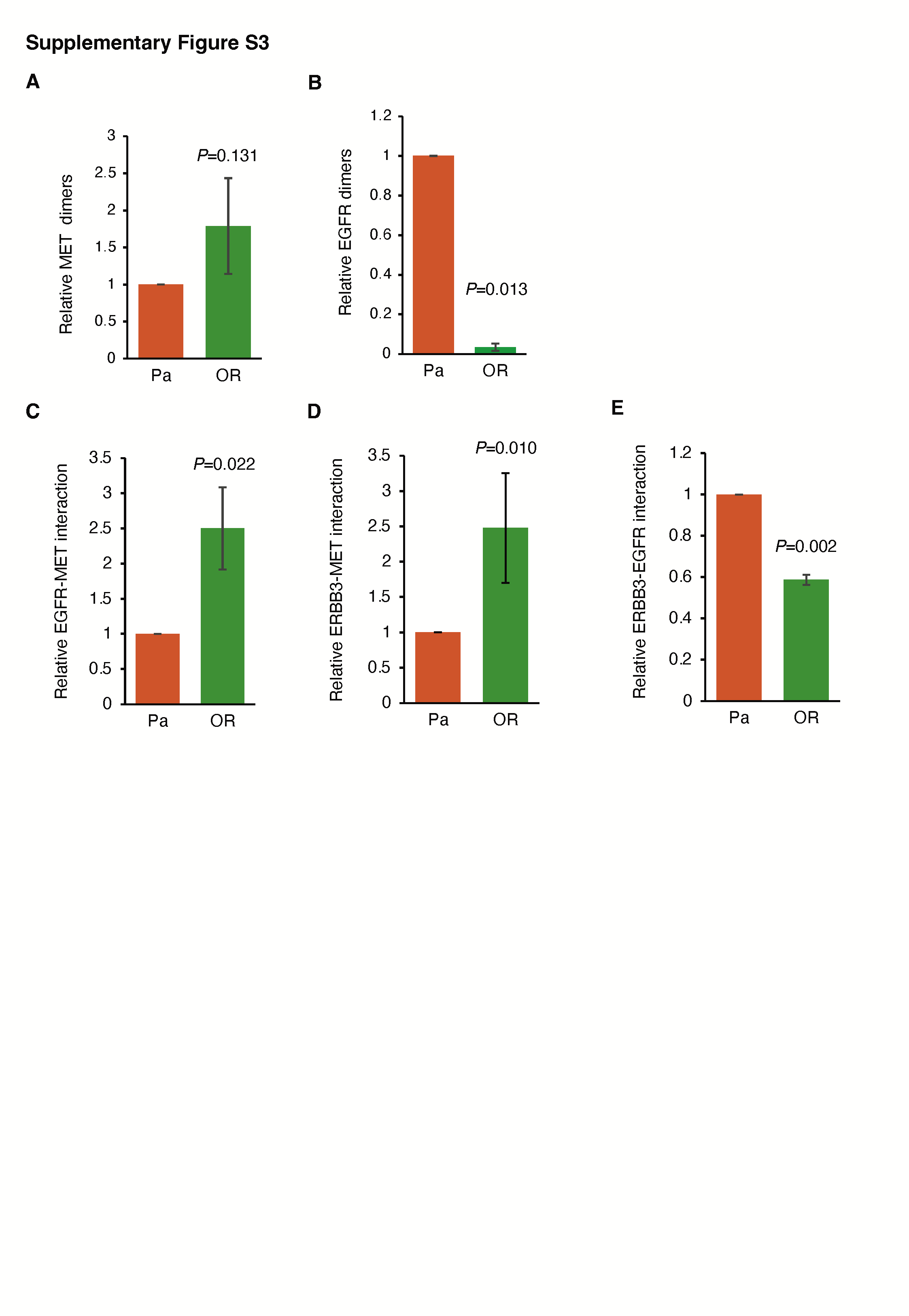

Supplement: Supplementary file 3 — Figure S3: Relative amounts of MET dimers (A) and EGFR dimers (B) in HCC827OR cells compared with HCC827Pa cells were quantified from immunoblots obtained with or without chemical crosslinking (n = 3, respectively). Relative EGFR‐MET (C), ERBB3‐MET (D), and ERBB3‐EGFR (E) interactions in HCC827OR cells compared with HCC827Pa cells were quantified from coimmunoprecipitation immunoblots (n = 3, respectively). [file TCA-17-e70334-s004.tiff]

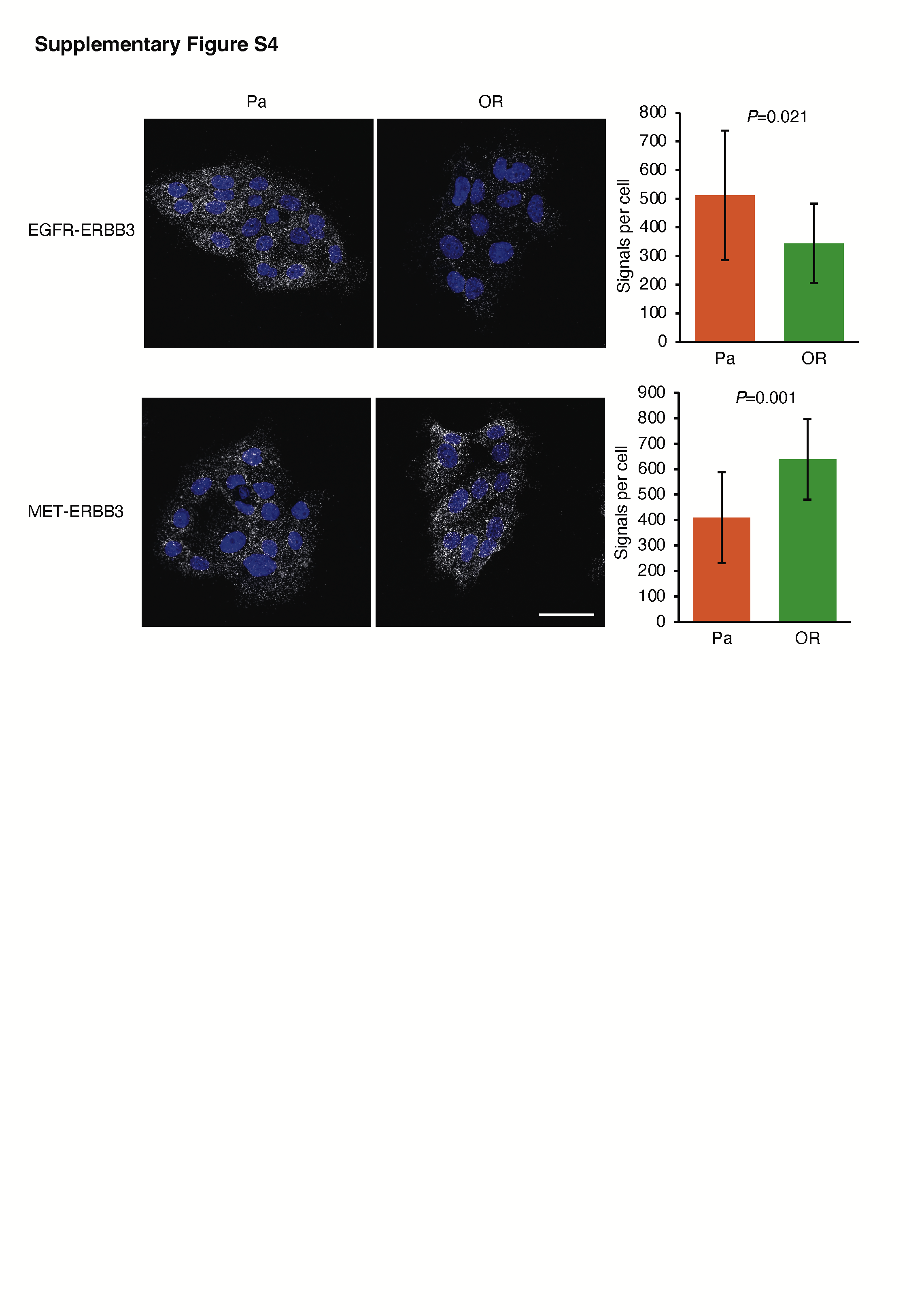

Supplement: Supplementary file 4 — Figure S4: Cells were subjected to PLA (left) with indicated pairs of antibodies (gray). Nuclei were stained with DAPI (blue). Representative images from one of 3 independent replicates are shown. Scale bar: 50 μm. Average numbers of punctate PLA signals per cell were counted from 5 images, and plotted in the graphs (right). [file TCA-17-e70334-s001.tiff]
